# A Bulwark of Security for the Patient

**Published:** 1919-09-27

**Authors:** 


### THE VOLUNTARY HOSPITAL ESSENTIAL TO PUBLIC SAFETY.

### III. A Bulwark of Security for the Patient.

It is becoming increasingly the fashion of very many people in a hurry, to cry out for Government Aid and Control in nearly everything at the present time. In this way a section of the public, moved by agitators and ill-informed persons, are in danger of being led into a blind alley, which may result in evils, dangers, and discomforts, from which all patients, who have the privilege of admission and treatment in the voluntary hospitals throughout Great Britain, are safeguarded. British hospitals are famed the world over as institutions where the patient's interests, welfare, and protection constitute the first consideration of everybody connected with these hospitals, from the highest to the lowest. In no other system of hospitals can the same claims be guaranteed to the same extent that they are in the best voluntary hospitals. From admission to discharge the patient, whether man, woman, or child, who is admitted to a voluntary hospital of the best kind, cannot properly have the slightest anxiety or doubt as to the character of the conditions, surroundings, and treatment which is there guaranteed for all the patients under treatment. Unfortunately, amazing as it may seem to those who are most intimately familiar with the administration of British hospitals of the best, the actual conditions and methods pursued in regard to patients at German and other hospitals, and the abuses often attaching to treatment within them, are neither appreciated nor known to the general public in this country, who are most dependent upon hospital care and treatment when necessity arises. It may fulfil a useful purpose to recall attention to a few facts relating to the treatment, handling, and discipline attaching to patients in German and other hospitals, as we have found them or they have been reported to us by those who have had actual experience of these matters.

### Consequences of an Evil Discipline.

We recently republished a guarded account of the evils and consequences of a rigid discipline and the absence of an exhibition of due regard to the feelings and rights of the patients under treatment, both in Berlin, Vienna, and in other foreign hospitals. The efficiency of any system, however admirable, must depend largely upon the spirit which dominates the individual men or women who bear the chief responsibility for the management and the conduct of its affairs. In Germany, before the war, there was markedly in many hospitals a much less keen sense of tenderness towards the sick, and indeed towards each other, than we, or the majority of us, in Great Britain and the United States enforce. The demands of science entail discipline upon the patients and the speedy carrying out of whatever has to be done in connection with the treatment. The enforcement of discipline, which is the keynote of the whole system of relations between the father and his family, the chief and his subordinates, emphasises the responsibility which attaches to individual heads of each department in German and Austrian hospitals. The deference displayed to the professor before the war by all workers in his laboratories in Germany was extraordinary. All this subordination to the chief was made even more striking and complete by the adoption of an identical attitude by the principal members of the professor's whole staff.

### The Position of the Patient.

In such circumstances the treatment of a patient, whether it be in the operation theatre or in any other department of the hospital, must largely depend upon the attitude of mind, the character and the principles of each chief of department, throughout a great establishment, where there may sometimes be met with as many as 2,000 patients, with the huge staff required to minister to their necessities. In our inspections the apprehension of these conditions and the marked and kindly forethought of some professors made us the better realise the actual condition of each patient. The German hospitals, as a class, have not contained anyone, as in the voluntary hospitals in England and the United States, whose special privilege and duty it is to consider the interests of the patients all the time, and to minister tenderly to the comfort of each. Scientific treatment abroad is apt to control affairs completely, to the setting back of humanity and the infinite lessening of the humanising influences which the spirit of personal service has brought about, as witness the delightful atmosphere to be found in the best administered voluntary hospitals in Great Britain, and some of the most admirable hospitals in the United States of America. Graduates who have studied in the hospitals and medical centres of Germany and Austria can testify, as we can from observation, that

Previous articles appeared on Sept. 13, p. 589, and 20, p. 611.

**The Voluntary Hospital Essential**—(*continued*).

in practice, where science comes first, and dominates the whole spirit of the administration of a great medical cure-house, there it will frequently be found that the spirit of personal service and infinite tenderness and human sympathy for the individual patient cannot, and does not, continually prevail.

The Responsibility of Silence.

The responsibility of silence placed upon the staff and the workers within British hospitals is a grave matter indeed, as we have often urged. State and principal hospitals, as at present conducted, abroad especially, too often do not sufficiently safeguard the interests or consider the natural feelings of the patients. It is this system and its freedom from independent criticism which make possible grave abuses without remonstrance or check. We are bound to testify that English men and women would never rest content to be patients in the general wards of institutions under an iron discipline which deprives the patients of the safeguards of an independent and articulate public opinion, which ministers to the setting back of humanity and the infinite lessening of the humanising influences which, without the spirit of personal service and trained nurses, cannot be maintained.

Every Sick Person in Hospital Walls.

It follows, and we wish to enforce this point with all possible authority and earnestness upon the people who to-day in England are so ready to cry out for the establishment of State hospitals, or rather the conversion of the voluntary hospitals into State hospitals, that the maintenance and extension of voluntary hospitals throughout the British Islands, if British people could only be brought to know and realise this, ministers to public safety, and the best interests of every sick person within hospital walls.

Foreign Government and Municipal Hospitals.

A personal inspection of the most representative Government and municipal hospitals Germany contained before the war, demonstrated that the technical standard of German hospitals was high, and tended constantly to become higher still. On these points there was small room for any differences of opinion, but prospective hospital patients in this country and elsewhere should realise and understand that, when all this had been said, and the fullest recognition had been given to the relatively high technical efficiency of the best hospitals of Germany at that time, we were bound to testify that English men and women would never rest content to be patients in the general wards of these institutions. They were often maintained under an iron discipline which deprived the patients of the safeguards of an independent and articulate public opinion.

Further, this iron discipline ministered to the setback of humanity and the infinite lessening of humanising influences, without which the spirit of personal service and trained nurses cannot be maintained. Where this service is present should be found the delightful atmosphere of our best administered voluntary hospitals in Great Britain and the United States. We confess to the strong sense of responsibility which we feel attaches to those who know the actual facts, for it follows that the more knowledge the people of these islands gain of a patient's life in Government and municipal hospitals conducted on the foreign principle, where scientific treatment is apt to control affairs completely, the more certain is it that a great system of State and municipal hospitals will never be set up in Great Britain, to the exclusion or destruction of the voluntary hospitals, which confer such infinite blessings upon all classes at the present time.

A Wise and Useful Reminder.

It would be a wise and useful reminder if every one throughout Great Britain were to have a card suspended in a prominent place in their cottage, living-room or flat, bearing the simple words:

**The Maintenance of the Voluntary Hospital System is Essential to the Well-being and Safety of all Classes.**

In concluding this series of three articles on certain aspects of the voluntary hospital which appear in these columns on September 13, September 20, and September 27, we hope for the co-operation of our contemporaries of all shades of opinion to make known the very important differences between the best voluntary hospitals with the principles on which they are conducted, and the dangers, disadvantages, and risks which often have attached to treatment in State and municipal hospitals abroad. Once lose the realisation of the continuous sense of our duty to our neighbour, and there might speedily attach to every hospital in the world (in the absence of the enforcement of the principles underlying the rights of every patient when admitted to it for treatment) the grave abuses which have been found in practice where science is the main, if indeed it is not often the paramount, consideration which has dominated the conduct of hospitals in the countries where, to the observant, they have made themselves notorious in the past.

A Glorious Privilege.

To be a Briton, an English man, woman or child is indeed a glorious privilege, a great honour, a grave responsibility, and our noblest heritage. It guarantees as the right of every one of us the maximum of security, and the tenderest and most considerate treatment in a voluntary hospital for one and all, when suffering from accident or overtaken by illness, disease, or injury which may necessitate operative or other grave risks, in an attempt to prolong life or preserve the continuous blessings of recovered health. These are the birthrights of every free man, woman, and child in our midst. May the League of Nations result in identical privileges, and their enforcement in practice for all members of the human family throughout the civilised world.

